# A Case-Control Seroprevalence Survey of Toxoplasmosis in Hemodialysis Patients and Healthy Subjects in Kazeroon and Jahrom Districts in Fars Province, Southern Iran

**DOI:** 10.1155/2023/8251462

**Published:** 2023-09-30

**Authors:** Shahin Kadkhodaei, Zahra Kargar Jahromi, Ali Taghipour, Hassan Rezanezhad, Kavous Solhjoo

**Affiliations:** ^1^Zoonoses Research Center, Jahrom University of Medical Sciences, Jahrom, Iran; ^2^Department of Medical Parasitology and Mycology, School of Medicine, Jahrom University of Medical Sciences, Jahrom, Iran

## Abstract

Opportunistic parasites such as *Toxoplasma gondii* (*T. gondii*) are capable of causing neurological and ocular manifestations in patients undergoing hemodialysis. By designing a matched case-control study, we conducted a seromolecular survey of *T. gondii* in hemodialysis patients compared to a healthy group from Jahrom and Kazeroon cities in Fars Province, Iran. For this purpose, 75 hemodialysis patients from Kazeroon city, 75 hemodialysis patients from Jahrom city, and 75 healthy individuals were recruited for the study. The serum levels of specific immunoglobulins (IgG/IgM) in the case and control groups were evaluated using the enzyme-linked immunosorbent assay (ELISA) method. Also, buffy coat samples were used to extract genomic DNA. Then, Polymerase Chain Reaction (PCR) was accomplished using the RE and GRA6 genes of *T. gondii*. A standard questionnaire containing demographic factors was administered. Although the seroprevalence of the anti-*T. gondii* IgG antibody in hemodialysis patients from Kazeroon (18.66% (14/75)) and Jahrom (25.33% (19/75)) was higher than that in the control group (13.33% (10/75)), no statistically significant difference was observed between the case and control groups (*P* value = 0.373 from Kazeroon and *P* value = 0.354 from Jahrom). Among the studied variables, only residence (urban) was significantly associated with the anti-*T. gondii* IgG antibody in the case group from Kazeroon. Also, no IgM antibody titers and DNA of *T. gondii* were detected in the case and control groups from both cities. Although high seroprevalence of anti-*T. gondii* IgG antibody was seen in hemodialysis patients, further epidemiological studies with larger samples need to be done in Jahrom and Kazeroon cities and in other parts of Iran. It is also necessary for health officials to establish programs for the prevention and control of *T. gondii* infection in hemodialysis patients.

## 1. Introduction

*Toxoplasma gondii* (*T. gondii*) is an opportunistic and zoonotic protozoan from the Apicomplexa phylum [1–3]. It is estimated that this pathogen affects almost a third of the world's population, especially in disadvantaged communities [4–6]. The life cycle of *T. gondii* consists of felines as definitive hosts and various vertebrates as intermediate hosts [7, 8]. Among the different hosts, only felines are able to contaminate environmental resources by excreting oocysts [9, 10].

Human toxoplasmosis is caused by ingesting soil, vegetables, or drinking water contaminated with sporulated oocysts [11] or undercooked or raw meat products containing viable tissue cysts [12]. Also, *T. gondii* may be crossed by transplacental transfer, blood transfusions, or organ transplantation [13–16] and mechanically spread by invertebrates (e.g., dung beetles, cockroaches, and earthworms) [17]. This pathogen is frequently asymptomatic/oligosymptomatic in immunocompetent people, but the protozoan may cause neurological and ocular manifestations in fetuses and immunocompromised individuals [18, 19].

Polymorphonuclear leukocyte dysfunction occurs due to increased levels of uremic toxins in patients with chronic kidney disease or the end stage of kidney disease [20]. As a result, the susceptibility to bacterial, viral, fungal, and parasitic infections is higher in these patients, so that infectious diseases are the second most prevalent cause of mortality in patients with end-stage renal disease undergoing hemodialysis [21, 22]. Many reports have shown that hemodialysis patients have a weak immune system in response to infectious agents, and as a result, these patients are susceptible to various opportunistic infections such as *T. gondii* [21, 23]. The current case-control study was performed to assess the seromolecular prevalence, sociodemographic, and risk factors associated with this parasite in hemodialysis patients.

## 2. Materials and Methods

### 2.1. Study Population and Sample Collection

To conduct this study, ethical approval was received from the Ethics Committee of Jahrom University of Medical Sciences, Jahrom, Iran (IR.JUMS.REC.1399.089). Sample collection was conducted during January 2020 to May 2020 from hemodialysis patients in the laboratories of the medical centers of Jahrom and Kazeroon cities in Fars Province, Iran. Considering the criteria, confirmed hemodialysis patients by the physician and its confirmatory tests and consent for participating were considered as criteria for the case group. Also, absence of kidney diseases, immune system defects, and those who are not under hemodialysis were the criteria for the control group. The control group was outpatients from other departments of medical centers in Jahrom and Kazeroon cities. Matching the age and sex of the control group with the case group was done for the accurate evaluation of the study. The sample size was calculated for the case and control groups using G^∗^Power software with a confidence level of 95%, type I error-*α* of 0.05, and power of the test (1-*β*) of 90%. Seventy-five subjects were calculated for each group (75 cases for Jahrom, 75 cases for Kazeroon, and 75 cases for the control group). A consent form and a standard questionnaire were organized for all subjects. Afterwards, about 10 cc of blood samples with/without EDTA was collected from each person. At first, after a rapid HIV test, people who were HIV negative were included in the study. Next, the collected samples were transported to the laboratory by maintaining cold chain conditions. To separate serum and buffy coats, blood samples were centrifuged at 600 × g for 10 minutes [24]. Lastly, EDTA-free serum for serological evaluation and buffy coats containing EDTA for molecular evaluation were preserved at -20°C until examinations.

### 2.2. Serological Assessments

Using enzyme-linked immunosorbent assay (ELISA) kits (Pishtazteb Co., Iran), serum samples were evaluated for anti-*Toxoplasma* IgG and IgM antibodies according to the manufacturer's protocols.

### 2.3. Molecular Evaluation

We extracted genomic DNA from buffy coat samples using the CinnaPure DNA extraction kit (CinnaGen Co., Tehran, Iran), as described in the manufacturer's instructions. RE and GRA6 genes were used for molecular evaluation. The selection of primer pairs, temperature setting, and times of each PCR reaction were adjusted according to our previous study [16]. Finally, PCR products for both RE and GRA6 genes were electrophoresed on a 1.2% agarose gel with SYBR® Safe stain and visualized using a UV transilluminator.

## 3. Results

A total of 225 subjects were included in the present study. Among these, 75 hemodialysis patients from Kazeroon city, 75 hemodialysis patients from Jahrom city, and 75 controls were involved. In terms of residence, 60% (45/75) of the subjects in Kazeroon and 65.3% (49/75) of the subjects in Jahrom were from urban regions. In terms of education, 16% (12/75) in Kazeroon and 30.66% (23/75) in Jahrom had a diploma and went to college. As shown in Tables 1 and 2, most of the participants were in the age range of 21-70+ years. It should be noted that we considered contact with cats as a risk factor, but none of the participants were in contact with them. More information of *T. gondii* seroprevalence among case and control individuals from Kazeroon and Jahrom is summarized in Tables 1 and 2, respectively.

The seroprevalence of anti-*T. gondii* IgG antibody in hemodialysis patients from Kazeroon (18.66% (14/75)) and Jahrom (25.33% (19/75)) was higher than that in the control group (13.33% (10/75)), and no statistically significant difference was observed between the case and control groups (*P* value = 0.373 from Kazeroon and *P* value = 0.354 from Jahrom). As shown in Table 1, only the residence (urban) was significantly associated with the anti-*T. gondii* IgG antibody in the case group from Kazeroon.

It should be noted that no IgM antibody titers were observed in the case and control groups from both cities. Considering PCR assays with the RE and GRA6 genomic target, no *T. gondii* bands were observed in the case and control groups from both cities.

## 4. Discussion

The purpose of this case-control study is to survey the seromolecular prevalence of *T. gondii* and its correlation with demographic and risk factors in hemodialysis patients in Kazeroon and Jahrom cities, Iran. In the present study, although a high seroprevalence of anti-*T. gondii* IgG antibody was observed in hemodialysis patients compared to the control group, this difference was not significant. In 2018, a national meta-analysis reviewing 10 articles showed a positive association between exposure to *T. gondii* and hemodialysis [23]. In this regard, the meta-analysis outcomes revealed that there is a significant OR between the increased prevalence of *T. gondii* exposure and hemodialysis (OR = 2.04; 95%CI = 1.54–2.70). In future studies, it is recommended to conduct more epidemiological studies of *T. gondii* in hemodialysis patients to gain a deep understanding of its status in this high-risk group.

As shown in Tables 1 and 2, we collected sociodemographic characteristics and risk factors in both case and control groups from both cities. Although only residence (urban) was significantly associated with the anti-*T. gondii* IgG antibody in the case group from Kazeroon, other variables should not be ignored. In this regard, in future studies, some factors such as contact with cats, consumption of raw vegetables, and contact with soil should be taken into consideration.

In the present study, no IgM antibody titers and DNA of *T. gondii* were observed in the case and control groups from both cities. It has been clearly demonstrated that individuals with anti-*T. gondii* IgG antibodies (lacking IgM) have chronic infection, but the presence of IgM does not definitively indicate acute infection [25]. Among the most important reasons for the difference in the prevalence rate of serological and molecular methods is the difference in their sensitivity and specificity [26]. However, PCR with its good sensitivity seems to be suitable for detecting circulating DNA in people with chronic toxoplasmosis [26]. Although the molecular results for *T. gondii* status in hemodialysis patients were negative, it is suggested that further molecular epidemiology with a higher sample size be performed in this population to better understand the *T. gondii* status and determine its genotypes.

## 5. Conclusion

These findings confirm a high prevalence of anti-*T. gondii* IgG antibody in hemodialysis patients. These patients are a risk group for *T. gondii* infection. Moreover, it is recommended that hemodialysis patients who are susceptible to *T. gondii* infection should be identified by serological tests of anti-*T. gondii* IgG and IgM antibodies. More epidemiological studies with larger samples need to be done in other parts of Iran, and there is a need for health officials to establish insight and control programs for *T. gondii* infection in hemodialysis patients.

## Figures and Tables

**Table 1:** Sociodemographic and risk factors of *Toxoplasma gondii* seroprevalence among hemodialysis patients in Kazeroon city.

Characteristic		IgG (case group)				*P* value	IgG (control group)				*P* value
		Positive		Negative			Positive		Negative		
		Frequency	%	Frequency	%		Frequency	%	Frequency	%	
Gender	Male	10	22.2	35	77.8	0.333	6	15	34	85	0.742
	Female	4	13.3	26	86.7		4	11.4	31	88.6	

Married	No	0	0	12	100	0.107	1	16.7	5	83.3	1
	Yes	14	22.2	49	77.8		9	13	60	87	

Age (year)	<20	0	0	3	100	0.568	0	0	1	100	0.539
	21-30	1	33.3	2	66.7		0	0	1	100	
	31-40	0	0	4	100		1	11.1	8	88.9	
	41-50	0	0	7	100		0	0	16	100	
	51-60	4	17.4	19	82.6		5	23.8	16	76.2	
	61-70	7	25.9	20	74.1		2	12.5	14	87.5	
	>70	2	25	6	75		2	18.2	9	81.8	

Education	Illiterate	4	13.3	26	86.7	0.654	3	7.3	38	92.7	0.723
	Primary school	1	7.1	13	92.9		5	45.5	6	54.5	
	Secondary school	4	21.1	15	78.9		0	0	10	100	
	Diploma	5	50	5	50		1	12.5	7	87.5	
	College	0	0	2	100		1	20	4	80	

Occupation	Housewife	5	15.2	28	84.8	0.573	3	9.1	30	90.9	0.452
	Self-employed	9	22.5	31	77.5		4	13.8	25	86.2	
	Employee	0	0	2	100		3	23.1	10	76.9	

Residence	Urban	12	26.7	33	73.3	0.029^∗^	6	11.3	47	88.7	0.467
	Rural	2	6.7	28	93.3		4	18.2	18	81.8	

^∗^Statistically significant difference (*P* value of <0.05).

**Table 2:** Sociodemographic and risk factors of *Toxoplasma gondii* seroprevalence among hemodialysis patients in Jahrom city.

Characteristic		IgG (case group)				*P* value	IgG (control group)				*P* value
		Positive		Negative			Positive		Negative		
		Frequency	%	Frequency	%		Frequency	%	Frequency	%	
Gender	Male	14	26.4	39	73.6	0.599	6	15	34	85	0.742
	Female	5	21.7	17	78.3		4	11.4	31	88.6	

Married	No	0	0	6	100	0.327	1	16.7	5	83.3	1
	Yes	19	27.5	50	72.5		9	13	60	87	

Age (year)	<20	0	0	1	100	0.596	0	0	1	100	0.539
	21-30	1	50	1	50		0	0	1	100	
	31-40	0	0	1	100		1	11.1	8	88.9	
	41-50	3	37.5	5	62.5		0	0	16	100	
	51-60	3	13.6	19	86.4		5	23.8	16	76.2	
	61-70	4	21.1	15	78.9		2	12.5	14	87.5	
	>70	8	36.4	14	63.6		2	18.2	9	81.8	

Education	Illiterate	7	29.2	17	70.8	0.666	3	7.3	38	92.7	0.723
	Primary school	4	17.4	19	82.6		5	45.5	6	54.5	
	Secondary school	1	20	4	80		0	0	10	100	
	Diploma	5	29.4	12	70.6		1	12.5	7	87.5	
	College	2	33.3	4	66.7		1	20	4	80	

Occupation	Housewife	3	15.8	16	84.2	0.088	3	9.1	30	90.9	0.452
	Self-employed	16	32	34	68		4	13.8	25	86.2	
	Employee	0	0	6	100		3	23.1	10	76.9	

Residence	Urban	15	30.6	34	69.4	0.110	6	11.3	47	88.7	0.467
	Rural	4	15.4	22	84.6		4	18.2	18	81.8	

^∗^Statistically significant difference (*P* value of <0.05).

## Data Availability

All data during the study are included in this manuscript.
